# Multidimensional fatigue and its impact on work productivity, mood and quality of life in long-term survivors following definitive intensity-modulated radiotherapy for oropharyngeal cancer: A cross-sectional study

**DOI:** 10.1007/s11764-024-01735-8

**Published:** 2025-01-18

**Authors:** Zsuzsanna Iyizoba-Ebozue, Emma Nicklin, Stuart Currie, James Price, J. P. C. Baldwin, Robin Prestwich, Sarah Brown, Emma Hall, John Lilley, Matthew Lowe, David J. Thomson, Finbar Slevin, Marcus Tyyger, Louise Murray, Florien Boele

**Affiliations:** 1 Department of Clinical Oncology, Leeds Cancer Centre, St. James’s University Hospital, Level 4, Bexley WingBeckett St., Leeds, LS9 7TF UK; 2 Leeds Institute of Medical Research at St James’s, University of Leeds, Leeds, UK; 3 Department of Neuroradiology, Leeds Teaching Hospitals NHS Trust, Leeds, UK; 4 Department of Clinical Oncology, The Christie NHS Foundation Trust, Manchester, UK; 5 Department of Medical Physics and Engineering, Leeds Teaching Hospital, Leeds, UK; 6 Leeds Cancer Research UK Clinical Trials Unit, Leeds Institute of Clinical Trials Research, Leeds, UK; 7 The Institute of Cancer Research, London, UK; 8 Department of Radiotherapy Physics, Leeds Cancer Centre, Leeds, UK; 9 Christie Medical Physics and Engineering, The Christie NHS Foundation Trust, Manchester, UK; 10 Faculty of Biology, Medicine and Health, Manchester Academic Health Sciences Centre, The University of Manchester, Manchester, UK; 11 Academic Unit of Health Economics, Leeds Institute of Health Science, University of Leeds, Leeds, UK

**Keywords:** Oropharyngeal cancer, Chronic fatigue, Late effects, Survivorship, Radiotherapy, Human papilloma virus

## Abstract

**Purpose:**

Radiotherapy (RT) for oropharyngeal cancer (OPC) can lead to late toxicity. Fatigue is a known debilitating issue for many cancer survivors, yet prevalence and severity of long-term fatigue in patients treated for OPC is unknown.

**Method:**

As part of a mixed-methods study, fatigue in OPC patients ≥ 2 years post RT + / − chemotherapy was evaluated. Fatigue scores (multidimensional fatigue inventory; MFI) were compared to general population controls. Predictive sociodemographic/clinical factors of fatigue were investigated by multivariable linear regression. Associations between fatigue, health related quality of life (EQ-5D-5L), work (work productivity and activity impairment — WPAI), mood disturbance (Profile of Mood Scale — POMS) and RT dose were explored.

**Results:**

In 349 patients treated for OPC with median follow-up time post-RT (+ / − chemo) of 6 years (*IQR* 4–8), > 20% reported severe fatigue in all domains. Scores were significantly worse in patients for mental (mean difference 1.2, 95% *CI* 0.6–1.8, *p* = < 0.001) and general fatigue (mean difference 0.8, 95% *CI* 0.1–1.3, *p* = 0.015) compared to controls. Age and co-morbidities were significant predictors of mental and general fatigue (*p* < 0.05). Worse fatigue was associated with worse quality of life, greater work productivity impairment and worse mood (*r* = − 0.604, 0.582 and 0.679, respectively, all *p* < 0.05). No correlation was found between fatigue and RT dose to the posterior fossa.

**Conclusions:**

Mental and general fatigue remain significant issues in OPC patients several years after RT + / − chemotherapy.

**Implications for Cancer Survivors:**

Better monitoring of fatigue throughout follow-up care, and timely interventions could help improve patient functioning.

**Supplementary Information:**

The online version contains supplementary material available at 10.1007/s11764-024-01735-8.

## Introduction

The treatment of oropharyngeal cancer (OPC) with (chemo-)radiotherapy may result in both physical and psychological late effects [1–3]. Survivorship research in patients treated for OPC, has focused primarily on head and neck-specific functional deficits such as impairments in swallowing and speech [4, 5], with need for further research evaluating complex multimodal issues such as fatigue.

The National Comprehensive Cancer Network (NCCN) defines cancer related fatigue as “a distressing, persistent, subjective sense of physical, emotional, and/or cognitive tiredness or exhaustion related to cancer or cancer treatment that is not proportional to recent activity and interferes with usual functioning” [6]. Fatigue may occur during cancer diagnosis, treatment and throughout the survival trajectory. It is considered one of the most prevalent and debilitating effects of cancer treatment. Fatigue is expressed through different behavioural outputs: physical (decreased energy level and reduced activity), mental (decreased ability to concentrate or attention) and affective (decreased motivation or interest) [7–9]. Consequently, fatigue levels may be influenced by many factors including cancer-related (e.g. pain, cancer treatment and anaemia) [10, 11] and non-cancer-related (e.g. lifestyle factors, sleep hygiene, anxiety and comorbidities) [11, 12], reflecting the complex and multidimensional nature of fatigue. Fatigue has been linked to lower health-related quality of life (HRQoL) [13–15], reduced work performance [16] and depression [13, 17] in other cancer survivors.

Confounding effects arising from the diverse combinations of treatments administered [18, 19], coupled with uncertainties surrounding long-term functioning, limit our understanding of long-term fatigue following non-surgical treatment for OPC. Therefore, this study aimed to evaluate fatigue as a multidimensional construct from 2 years post-RT. Furthermore, we aimed to explore the relationship between fatigue and other patient-centred outcomes (HRQoL, mood and work performance), and to explore possible predictors for fatigue together with associations between fatigue and posterior fossa radiotherapy dose. Insights gained could help identify patients at risk of long-term fatigue, identify key areas for intervention and help guide development of comprehensive support services.

## Methodology

### Study design

This study was part of the cross-sectional, mixed methods, multicentre ROC-oN (Radiotherapy for Oropharyngeal Cancer and impact on Neurocognition) study, approved by the West Midlands Research Ethics Committee in October 2022 (22/WM/0207). We assessed patient-reported outcomes (reported here), as well as cognitive functioning (reported separately). A subset of participants were interviewed [20].

### Participants and treatment

Patients enrolled in this study were treated for OPC with primary RT (+ / − chemotherapy) at Leeds Teaching Hospitals NHS Trust (LTHT) or The Christie NHS Foundation Trust (The Christie) between 2010 and 2020. In these centres, prior to 2013, patients were treated with 3-dimensional conformal radiation therapy (3DCRT). Thereafter, intensity modulated radiation therapy (IMRT) and volumetric modulated arc therapy (VMAT) were used, with 5 fractions delivered per week with a dose per fraction of 2–2.17 Gy, with or without concurrent chemotherapy. Participant eligibility criteria were > 18 years old, ≥ 24 months follow-up and disease free at time of recruitment. Exclusion criteria were upfront or salvage surgery. Participants provided written informed consent.

### Outcome measures

Clinical data were extracted from medical records.

The brainstem and anterior and posterior cerebellum were evaluated as substructures of interest in patients treated in Leeds (*n* = 144). The rationale and process for this selection and delineation methods are described in supplementary (Sect. 1). Dose distributions were converted to equivalent doses in 2 Gy fractions assuming an α/β of 3 Gy [21] and for each substructure, the following dosimetric information was recorded: near-maximum dose (D1cc), mean dose (Dmean) and values for V10–V30 (in 5 Gy increments with 95% *CI*). To mitigate potential multicollinearity among CNS-ROIs, an additional composite structure termed the “posterior fossa” (comprising the brainstem, anterior and posterior cerebellum) was created, and the same dosimetric information was exported.

### Patient-reported outcomes

Patients completed self-administered questionnaires at a single time point. Patients provided information regarding sociodemographic details including age, sex, ethnicity, level of education, household income, marital status, employment status, comorbidities and smoking history.

*Fatigue* was assessed using the multidimensional fatigue inventory (MFI) [22–24]. The MFI is a 20-item measure yields five 4-item domain scores (score range 4–20 with higher scores indicating worse fatigue), with good internal consistency in our cohort [25]: general fatigue (Cronbach’s *ɑ* = 0.79, *n* = 340), physical fatigue (*ɑ* = 0.84), reduced activity (*ɑ* = 0.82), mental fatigue (*ɑ* = 0.81) and reduced motivation (*ɑ* = 0.69). A control group of individuals from the general UK population without a cancer diagnosis were used (data collected as part separate investigation to collect UK normative scores for the cognitive tests used in the parent study, not yet published), matched to our patient cohort for age and gender (*n* = 380).

*HRQoL* was evaluated using the EQ-5D-5L and visual analogue scale (EQ-VAS). The descriptive system assesses if there are no problems (score = 1), some problems (score = 2–4), or extreme problems (score = 5]) across 5 domains: mobility, self-care, usual activities, pain/discomfort and anxiety/depression [26]. The EQ VAS rates health on a vertical scale from 0 (“the worst health you can imagine”) to 100 (“the best health you can imagine”) [27–29].

*Mood* was measured with the Profile of Mood States short form (POMS-SF). The POMS-SF comprises 37 items (5-point Likert scale) making up 6 subscales [30]: tension, depression, anger, fatigue, confusion (higher scores are worse) and vigour (higher scores are better). The total mood disturbance (TMD) score is calculated, with higher scores indicating worse mood [31, 32].

*Work productivity* was assessed using the Work Productivity and Activity Impairment (WPAI), which provides a measure of total work productivity impairment (TWPI) and total activity impairment (TAI) in the past 7 days. TWPI accounts for absenteeism (away from work due to sickness or disability) and presenteeism (productivity loss even though at work due to underperformance as a result of sickness or medical conditions), while TAI measures limitations in carrying out unpaid activity due to health problems [33, 34]. The results are expressed as impairment percentages; higher values indicate less productivity, i.e. worse outcomes.

### Data analysis

Descriptive analysis was performed for sociodemographic and clinical data. Questionnaire data were handled as per scoring manuals. To meet the first study aim (to evaluate fatigue as a multidimensional construct in patients with OPC treated with radiotherapy), MFI domain scores were calculated and severity determined as follows: mild (5–8), moderate (9–12) and severe (13–20) [35] to enhance clinical utility of findings. A total score can be calculated (T-MFI, score range 20–100). Using the cancer-free controls from the general UK population matched for age and gender, *t*-tests were performed to compare mean scores. We hypothesized that fatigue would be worse in OPC survivors than in controls.

The second aim of this study was to explore the relationship between fatigue and other patient-centred outcomes as an indicator of the impact on everyday life. Spearman correlations were used to assess associations between the MFI domain scores and HRQoL (EQVAS), mood (POMS total score) and work performance (TWPI and TAI). Correlation coefficients between 0.8–0.9/–0.8 and –0.9 were classed as strong, 0.6–0.7/ − 0.6 and –0.7 as moderate, 0.3–0.5/–0.3 and –0.5 as fair and < 0.3/–0.3 as weak [36]. To process EQ-5D-5L data, the patient cohort was divided into 2 groups based on the descriptive system: a state of perfect health “no health problems” (11111 on all EQ-5D) and “health problems” (any health profile other than 11111) [37], with a one-way ANOVA performed to assess differences in fatigue outcomes. We hypothesized that worse fatigue would be associated with worse HRQoL, mood and work performance outcomes.

A third aim of the study was to identify potential predictors for fatigue in patients treated for OPC. We selected the MFI scale scores found to be significantly different from controls only. Associations between MFI scale scores and clinical factors (smoking, age, sex, ECOG performance status, number of co-morbidities and employment) disease (HPV, tumour subsite and TNM 7 stage) and treatment-related independent variables (radiation dose-fractionation, chemotherapy and time from end of treatment) were initially explored on univariate regression analysis. Here, no specific hypotheses were set as these analyses were explorative. Those variables associated with fatigue at *p*-value ≤ 0.1 were carried forward to multivariable linear regression. Multicollinearity between the variables was evaluated, and variables were excluded if the variance inflation factor (*VIF*) was ≥ 10. Backward elimination was performed on multivariable analysis. Coefficients and 95% confidence intervals were reported.

A fourth aim was to evaluate potential relationships between fatigue and the dose received by the posterior fossa structures. This dosimetric information was available only from patients recruited at LTHT (*n* = 144). Spearman correlation between fatigue domains (in which significant differences from normative scores noted) and dose (Dmax, Dmean and low dose bath) to CNS-ROIs evaluated. We hypothesized that worse fatigue would be associated with higher dose RT to the base of the brain.

Missing data were below 10% for each variable (4% for MFI, EQ-D5-5L, WPAI and 9% of POM-SF), except RT dosimetry which were not available for the Christie participants (57%) and were not confirmed missing completely at random (MCAR) and therefore were not imputed. Statistical significance was set at *p* < 0.05 for all tests, except for the univariate regression analyses described above. In line with the exploratory nature of the study and its specific aims, no corrections for multiple testing were applied in building the regression models. Data were analysed in STATA 18.0.

## Results

### Participants

An invitation to participate was sent out to 855 patients previously treated for OPC; 349 were enrolled, of whom 151 and 198 were treated at LTHT and The Christie, respectively. Sociodemographic and clinical characteristics are summarised in Table 1. The median time post-RT was 6 years (*IQR* 4–8 years). Annual household income was below the UK national average for 48.4% [36]; 60% were married and 69% co-habited with family.

**Table 1:** Participant, disease and treatment characteristics

	Patients treated for OPC (*n* = 349)	Control group (*n* = 380)
Age		
• Age at diagnosis median (*IQR*)	58.6 (52–64) years	N/a
• Age at study recruitment median (*IQR*)	64 (59–70) years	63 (54–70) years
• Age categories		
40–49	12 (3.4%)	51 (13.4%)
50–59	82 (23.5%)	84 (22.1%)
60–69	147 (42.1%)	146 (38.5%)
≥ 70	108 (31%)	99 (26%)
Sex		
• Male (%)	256 (73.4%)	228 (60.0%)
• Female (%)	93 (26.6%)	151 (40.0%)
Level of education		
• No formal educational	58 (16.9%)	3 (0.8%)
• No university degree	217 (63.3%)	217 (57.1%)
• University degree or higher	50 (14.5%)	160 (42.1%)
• Unknown	17 (4.9%)	0 (0.0%)
Current employment status		
• Employed	146 (41.8%)	
• Unable to work due to illness/disability	19 (5.4%)	
• Unemployed	8 (2.2%)	
• Retired	167 (47.8%)	
• Unknown	9 (2.5%)	
Pretreatment ECOG performance status		
• 0	300 (86.0%)	
• 1	45 (12.9%)	
• 2	4 (1.1%)	
Number of co-morbidities		
• None	115 (32.9%)	
• 1	122 (34.9%)	
• 2	62 (17.7%)	
• 3/ > 3	44 (14.3%)	
• Unknown	6 (1.7%)	
Smoking at time of treatment		
• Current smoker	65 (18.6%)	
• Ex-smoker	114 (32.6%)	
• Never smoked	165 (47.2%)	
• Unknown	5 (1.4%)	
Tumour subsite		
• Tonsil	217 (62.2%)	
• Base of tongue	116 (33.2%)	
• Vallecula	2 (0.6%)	
• Posterior pharyngeal wall	6 (1.7%)	
• Soft palate	8 (2.3%)	
T-stage		
• T1	65 (18.6%)	
• T2	166 (47.5%)	
• T3	63 (18.0%)	
• T4	55 (15.7%)	
N-stage TNM7		
• N0	55 (15.8%)	
• N1	44 (12.6%)	
• N2	208 (59.6%)	
• N3	42 (12%)	
P16 status		
• Positive	286 (81.9%)	
• Negative	26 (7.4%)	
• Unknown	37 (10.6%)	
Fractionation		
• 70 Gy/35#	142 (40.6%)	
• 65–66/30#	204 (58.4%)	
• 50 Gy/20#	3 (0.8%)	
Concurrent chemotherapy	279 (79.9%)	
Radiotherapy alone	70 (20.1%)	

*T-stage* tumour stage, *N-stage* nodal stage, *P16* human papilloma status, *ECOG* Eastern Cooperative Oncology Group performance status

### Fatigue as a multidimensional construct

**Fig. 1a:**
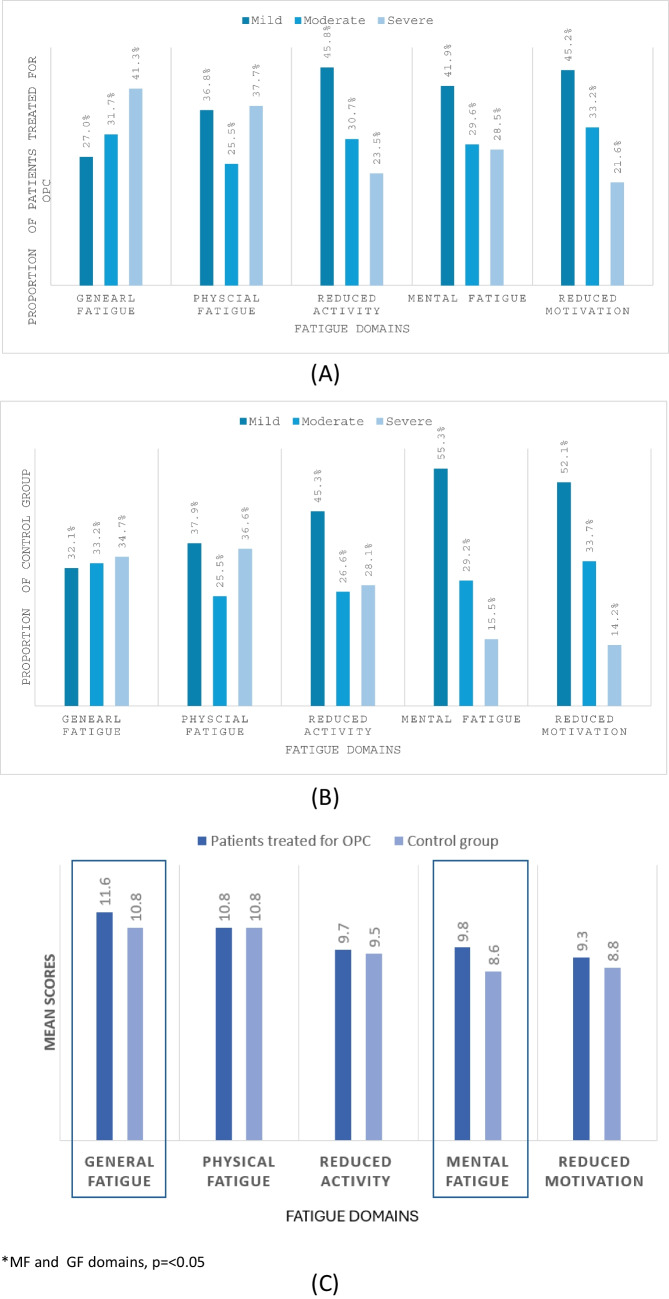
**A** Prevalence of fatigue across fatigue domains in patients treated for OPC (*n* = 349), **B** prevalence of fatigue across fatigue domains in control group (*n* = 380) and **C** mean scores in fatigue domains in people treated with OPC (*n* = 349) and control group (*n* = 380)

On the T-MFI, 31% reported severe fatigue (*MFI* > 60.5); 32% moderate (*MFI* 43.5–60.5) and 37% mild (*MFI* < 43.5). Severe fatigue was reported by over 20% of patients treated for OPC in every domain with severe fatigue predominantly reported in GF (41.3%) (Fig. 1A; Table 2). Mean scores for fatigue domains ranged from 9.39 (SD 4.29) to 11.62 (4.89) (Fig. 1B; Table 2). Statistical differences in mean score between patients treated for OPC and matched controls were observed in the domains of mental fatigue (mean difference 1.2, 95% *CI* 0.6–1.8, *p* = < 0.001) and general fatigue (mean difference 0.8, 95% *CI* 0.1–1.3, *p* = 0.015), indicating heightened levels of fatigue in these domains among patients treated for OPC (Fig. 1C).

**Table 2:** Patient reported outcomes

Patient outcomes	*N* (%)	*Mean* (*SD*)
*MFI*		
General fatigue		11.12 (4.89)
Physical fatigue		10.43 (5.10)
Reduced activity		9.18 (4.65)
Mental fatigue		9.46 (4.72)
Reduced motivation		8.99 (4.29)
*EQ-5D and EQVAS*		
* EQ-5D*		***EQVAS***
Perfect health	95 (27.2%)	88.02 (8.26)
Other Health	254 (72.85)	69.83 (19.36)
Perfect health in presence of mental fatigue		
Mild fatigue	59 (62.11%)	83.00 (15.85)
Moderate fatigue	23 (24.21%)	71.59 (18.14)
Severe fatigue	13 (13.68%)	67.10 (19.19)
Perfect health in presence of general fatigue		
Mild fatigue	46 (48.42%)	87.84 (10.24)
Moderate fatigue	34 (35.79%)	76.84 (17.07)
Severe fatigue	15 (15.79%	65.66 (19.66)
*POM-SF*		
Fatigue-inertia		6.17 (5.81)
Depression-dejection		4.17 (6.38)
Anger-hostility		3.20 (5.03)
Tension-anxiety		4.55 (5.59)
Confusion-bewilderment		3.45 (4.36)
Vigor-activity		9.74 (6.24)
Total mood disturbance		11.81 (25.67)
*WPAI*		
* Participants working*	146	
Percent work time missed due to ill health (those with missed time > 0)	20 (13.6%)	
Percent impairment while working due to health (those with % impairment while working > 0)	62 (42.5%)	
Overall percent work impairment (TWPI) (*mean* %)	-	19.69%
*All participants*		
Percent activity impairment due to health (TAI) (those with % activity impairment > 0)	210 (62.5%)	
Overall percent activity impairment (TAI) (*mean* %)	-	27.14%

*MFI* multidimensional fatigue inventory, *EQ-VAS* EQ-Visual Analogue Scale, *POM-SF* profile of mood short form, *WPAI* work productivity and activity impairment, *TWPI* total work productivity impairment, *TAI* total activity impairment

### Fatigue and HRQoL, mood and work productivity

Statistically significant negative correlations of fair to moderate strength were observed between fatigue domain scores and EQ-VAS scores, indicating that HRQOL declines with higher reports of fatigue (Table 3). A moderate positive correlation demonstrated that worse mood (total POMS score) was associated with fatigue (Table 3).

**Table 3:** Correlation coefficients between fatigue and outcomes (*all *p* < 0.001)

	General fatigue *r*	Physical fatigue *r*	Reduced activity *r*	Mental fatigue *r*	Reduced motivation *r*
EQ VAS	− 0.604	− 0.672	− 0.578	− 0.443	− 0.514
Total activity impairment (TAI)	0.606	0.686	0.573	0.468	0.529
Total work productivity impairment (TWPI)	0.582	0.572	0.377	0.436	0.409
Total mood disturbance (TMD)	0.679	0.602	0.532	0.609	0.595

In our sample, 146 (41.8%) patients treated for OPC were employed at the point of assessment. Within the subset of actively employed individuals, 142 were ≤ 65 years old. Overall, 70% of those below retirement age in the total sample (*n* = 349) were working. Presenteeism was reported in 42.5% and absenteeism reported 13.6%. Among all patients treated for OPC, > 50% reported some level of impairment in TAI (total activity impairment), with 27.9% of patients reporting activity levels impaired by ≥ 50%. Statistically significant positive correlations of fair to moderate strength were found between fatigue domain scores and TWPI as well as TAI, suggesting greater impairment in both paid and unpaid work with increasing fatigue severity (see Table 3).

### Fatigue and potential clinical predictors

Mental fatigue was associated with age at treatment, sex, smoking, number of co-morbidities and employment status on univariate regression analysis. In the multivariate analysis, age at treatment and the number of comorbidities were identified as key indicators. For each additional year of age at treatment, mental fatigue score decreased by 0.1 (coefficient − 0.1, 95% *CI* − 0.2 to − 0.05, *p* < 0.001), while each additional comorbidity was associated with a 0.6 increase in the mental fatigue score (coefficient 0.6, 95% *CI* 0.2 to 1.3, *p* = 0.002) (supplementary Sect. 2).

In univariate analysis, general fatigue was associated with age at treatment, number of co-morbidities and employment. Similar to mental fatigue, multivariate analysis revealed age at treatment and comorbidities as important predictors of general fatigue. Age at treatment showed a negative association (coefficient − 0.1, 95% *CI* − 0.2 to − 0.05, *p* < 0.001) and additional comorbidities increased general fatigue score by 1.1 (coefficient 1.1, 95% *CI* 0.7 to 1.8, p < 0.001) (supplementary Sect. 2).

Time since radiotherapy was not observed to have any statistically significant impact on mental fatigue (coefficient − 0.1, 95% *CI* − 0.3 to 0.1, *p* = 0.37) or general fatigue (coefficient − 0.1, 95% *CI* − 0.3 to 0.1, *p* = 0.33) scores.

### Fatigue and CNS substructure of interest radiotherapy doses

No significant correlations were found between dose to CNS-ROIs and long-term mental or general fatigue (*r* < 0.3, *p* > 0.05 for all). Dose received by the posterior fossa substructures presented in supplementary (Sect. 3).

## Discussion

Almost all cancer patients experience fatigue during active treatment; however, fatigue may persist into longer term survivorship. In our large sample of patients who were treated for OPC on average 6 years previously, > 20% experienced severe fatigue beyond 2 years post-radiotherapy (+ / − chemotherapy) in all domains. On a group level, significant differences were observed between patients treated for OPC and matched controls from the general population for mental and general fatigue domains. An increasing burden of fatigue was linked to diminished HRQoL and mood disturbance. As fatigue levels increased, work productivity declined. Although there is no established “minimally important difference” for MFI scores, our findings were backed up by the qualitative report from the same parent study. In in-depth interviews with 21 patients treated for OPC, the emotional and mental aspects of fatigue were similarly described as affected, leading to “a new normal” characterised by impaired work capacity, poor engagement with leisure activities, low mood and in some cases social isolation [20].

In a cross-sectional study, (*n* = 47), a negative correlation was found between HRQoL and fatigue perception 1 year after diagnosis of head and neck cancer (HNC) [38]. Huynh et al. [39] in a larger cross-sectional study (*n* = 227) of a heterogeneous group of survivors of HNC also found similar results several years following treatment (median follow-up time of 8.5 years) [39]. Our finding was consistent with this. The EORTC Core Quality of Life questionnaire (EORTC QLQ-C30) was developed to assess QoL in cancer patients and is used by most studies. However, we were able to demonstrate the inverse relationship between HRQoL and fatigue using the EQ-5D-5L. This could be due to a correlation between similar domains of the EORTC QLQ-C30 and EQ-5D-5L [40].

In a prospective study in patients receiving RT for HNC, fatigue was associated with depression during and immediately after RT [41]. It is recognised that 5 years after diagnosis, patients with HNC face a significantly elevated risk of developing depression [18]. In our sample, the increasing burden of general and mental fatigue was associated with greater mood disturbance. Although fatigue is part of the Diagnostic and Statistical Manual of Mental Disorders (DSM) criteria for major depressive disorder, some perceive fatigue and depression to represent separate symptoms among cancer survivors, exhibiting a nuanced overlap [15].

As fatigue levels increased among OPC patients in our sample, a decrease in work productivity was noted. Despite 70% of study participants within the working-age group being employed, presenteeism, characterized by underperformance at work, was identified as the primary cause of reduced productivity. Whilst difficulty returning to work is a known issue for HNC survivors [42–46], our findings highlight that also those able to return to work face challenges. Indeed, our qualitative report confirmed that even years after treatment, many individuals are only able to work reduced hours or need to change employment post-treatment [20]. This underscores the long-lasting impact of cancer on work ability, which has known implications for HRQoL, financial stability, self-identity and a sense of normalcy [47, 48].

Notably, age at treatment initiation and a higher number of comorbidities were factors independently associated with mental and general fatigue severity. The association between age at treatment and cancer-related fatigue lacks consensus [12, 14, 49, 50]. However, OPC and its treatment at a younger age may result in a greater perception of fatigue due to accelerated depletion of physiological and biological reserves [51], leading to premature ageing [52]. The presence of two or more comorbidities has been identified as a significant predictor of post treatment HRQoL in patients with HNC [19, 53, 54], which was supported by our study. Interestingly, whilst some evidence exists for acute fatigue following RT to the brainstem, anterior and posterior cerebellum [55–57], we were unable to confirm a link between RT dose to the base of the brain and long-term fatigue outcomes. In part, this discrepancy in findings may result from differences in RT dose, with a higher mean RT dose delivered to the posterior fossa in the PARSPORT trial (23–25 Gy in the IMRT-treated group vs 5 Gy in our cohort) [51]. Notably, our finding aligns with the only other study to investigate dose relationship with long-term fatigue in a heterogeneous cohort of HNC survivors [39]. Fatigue is a complex and multidimensional construct and can be influenced by multiple cancer and non-cancer related factors, not all of which we were able to capture in the present study (e.g. lifestyle factors and sleep hygiene). The differences observed between OPC survivors and controls from the general population who have never had a cancer diagnosis would suggest some impact of the diagnosis itself, perhaps irrespective of treatment.

Our study holds strengths in that it evaluates fatigue as a multidimensional construct in a large sample of OPC survivors several years after non-surgical treatment. The cross-sectional nature of the study is a limitation as it does not allow for evaluation of changes in fatigue over time. Whilst we compared fatigue levels between OPC survivors and a group of matched controls without a cancer diagnosis, it is unclear whether the statistically significant differences found reach a clinically significant level. However, the qualitative arm of this study strongly suggests it is [20]. Due to overlap in definitions and/or diagnostic criteria, HRQoL and depression outcome measures often include fatigue symptoms, which make finding associations with fatigue more likely — whilst we used EQ-5D-5L which does not include fatigue symptoms, the POMS-SF does cover fatigue. There is the potential for selection bias as approximately 41% of invited patients took part in the study (might reflect challenge of recruiting participants who are 5 or more years post treatment), and the sample includes predominantly patients with HPV-positive driven disease, limiting the generalisability of findings to survivors of HPV-negative OPC. Finally, outcomes such as sleep quality, fear of cancer recurrence, anxiety and social functioning [10, 58, 59] which may also influence fatigue, were not evaluated and should be considered in future investigations.

In conclusion, this study demonstrates the substantial burden of fatigue in patients on average 6 years after non-surgical OPC treatment, with associations to other patient-centred outcomes including HRQoL, mood and work productivity indicative of great impact on the daily lives of patients. Existing comorbidities and identified correlates are hypothesis generating for potential determinants of chronic fatigue in patients treated for OPC. Future work will include validating these findings in an independent external cohort to assess their generalisability and robustness. Finally, exercise has been found effective in reducing cancer-related fatigue, leading to its incorporation into management guidelines [60]. However, HRQoL in patients treated for OPC might also be improved through interventions aimed at alleviating mental fatigue such as mindfulness, stress reduction programmes, focusing on one task at a time with short work periods, prioritizing tasks, planning daily activities and avoiding over-exertion [61].

To improve patient wellbeing into longer term survivorship, we highlight the need to establish better follow-up care. This may include improved patient information, incorporating fatigue screening into routine practice, with closer monitoring in high-risk patients, and establishing effective (self-management) interventions to prevent or alleviate fatigue. Effective management of fatigue may substantially improve patient functioning and limit the adverse effects of an OPC diagnosis and its treatment on patients’ lives.

## Supplementary Information

Below is the link to the electronic supplementary material.

Supplementary file1 (DOCX 36 KB)

## Data Availability

No datasets were generated or analysed during the current study.
