# Supplementary material for: Multidimensional fatigue and its impact on work productivity, mood and quality of life in long-term survivors following definitive intensity-modulated radiotherapy for oropharyngeal cancer: A cross-sectional study
Source: J Cancer Surviv. 2025 Jan 18;20(3):1330–40. doi: 10.1007/s11764-024-01735-8 (PMC13144248; doi:10.1007/s11764-024-01735-8)
Supplement: Supplementary file 1 — Supplementary file1 (DOCX 36 KB) [file 11764_2024_1735_MOESM1_ESM.docx]

**Supplementary**

1. Dose work

Radiation dose to the central nervous system (CNS) has been postulated as a potential contributor to treatment-related fatigue, in head and neck cancer (HNC) patients following radiotherapy[1-3] . Hence association between radiation dose to CNS structure and long-term fatigue was evaluated. Dosimetric study was conducted as below

1. Phase I: An atlas of HNC CNS regions of interest (CNS-ROIs) was developed (ZIE,LM,SC) integrating brain sub-structures identified from existing literature and the European Particle Therapy Network (EPTN) atlas. CNS-ROIs included in the atlas were amygdala-hippocampus (contoured as one structure), brainstem, cerebellum (anterior & posterior contoured separately), temporal lobes (right and left contoured separately) and thalamus. RT treatment plans for participants treated in Leeds (n=144) were retrieved from the original RT planning system (Monaco, Elekta AB, Sweden) and restored in RayStation (V11A RaySearch Laboratories AB, Sweden), with diagnostic MRI co-registration where possible. The above CNS-ROIs were retrospectively manually delineated by a clinical oncologist (ZIE) in 40 cases and contours were quality assured by an experienced neuro-radiologist (SC). Dose distributions were examined across all structures and, to limit multiple hypothesis testing, only those structures considered to have received a meaningful radiotherapy dose (brainstem and anterior and posterior cerebellum) were included for further dosimetric analysis.
2. Phase II: The brainstem and anterior and posterior cerebellum were contoured on datasets for the remaining 104 participants from Leeds, again with MRI co-registration where possible. For each of these sub-structures, the following dosimetric information was recorded: near-maximum absorbed dose (D1cc), mean absorbed dose (Dmean), and values of V10-V30 (in 5Gy increments with 95% CI). To mitigate potential multicollinearity among CNS-ROIs, an additional composite structure termed the "posterior fossa" (comprising the brainstem, anterior, and posterior cerebellum) was created and the same dosimetric information exported.
3. Multivariate Regression Analysis Results: Examining relationship between Mental Fatigue and independent variables (only statistically significant results presented)
4. Mental fatigue

| Variable | Coefficient | Std Error | t | p value | 95% CI |
| --- | --- | --- | --- | --- | --- |
| Age | -0.10 | 0.02 | -3.77 | <0.001 | -0.16 **-** -0.05 |
| Number of comorbidities | 0.69 | 0.22 | 3.13 | 0.002 | 0.26  **-** 1.13 |

*(Adjusted R^2^ = 0.05, F (2, 330) = 10.2, p= <0.001)*

1. General fatigue

| Variable | Coefficient | Std Error | t | p value | 95% CI |
| --- | --- | --- | --- | --- | --- |
| Age | -0.10 | 0.02 | -3.82 | <0.001 | -0.15 **-** -0.05 |
| Number of comorbidities | 1.15 | 0.21 | 5.31 | <0.001 | 0.72  **-** 1.57 |

*(Adjusted R^2^ = 0.10, F (2, 339) = 18.4, p= <0.001)*

1. Dose data to CNS-ROIs

|  | Brainstem  (mean, SD) | Anterior Cerebellum  (mean, SD) | Posterior Cerebellum  (mean, SD) | Posterior Fossa  (mean, SD) |
| --- | --- | --- | --- | --- |
| Dose to 1cc (Gy) | 25.39 (7.38) | 3.68 (3.63) | 29.77 (8.67) | 30.38 (7.9) |
| Mean dose (Gy) | 2.05 (1.52) | 1.5 (1.04) | 7.15 (4.45) | 5.1 (3.6) |
| V10 (cm^3^) | 6.01 (2.79) | 0.2 (0.66) | 41.13 (24.21) | 43.25 (24.7) |
| V15(cm^3^) | 4.58 (2.46) | <0.1 | 23.25 (19.75) | 24.8 (19.5) |
| V20(cm^3^) | 3.02 (2.22) | <0.1 | 12.50 (14.31) | 13.6 (14.5) |
| V25(cm^3^) | 1.72 (1.67) | <0.01 | 6.12 (8.88) | 6.7 (8.9) |
| V30(cm^3^) | 0.77 (0.95) | <0.001 | 2.69 (4.92) | 2.8 (4.7) |

[1] Gulliford SL, Miah AB, Brennan S, McQuaid D, Clark CH, Partridge M, et al. Dosimetric explanations of fatigue in head and neck radiotherapy: an analysis from the PARSPORT Phase III trial. Radiotherapy and oncology : journal of the European Society for Therapeutic Radiology and Oncology. 2012;104:205-12.

[2] Ferris MJ, Zhong J, Switchenko JM, Higgins KA, Cassidy RJ, McDonald MW, et al. Brainstem dose is associated with patient-reported acute fatigue in head and neck cancer radiation therapy. Radiotherapy and Oncology. 2018;126:100-6.

[3] Abel E, Silander E, Nordström F, Olsson C, Brodin NP, Nyman J, et al. Fatigue in Patients With Head and Neck Cancer Treated With Radiation Therapy: A Prospective Study of Patient-Reported Outcomes and Their Association With Radiation Dose to the Cerebellum.
